# Supplementary material for: Differential chromatin binding of the lung lineage transcription factor NKX2-1 resolves opposing murine alveolar cell fates in vivo
Source: Nat Commun. 2021 May 4;12:2509. doi: 10.1038/s41467-021-22817-6 (PMC8096971; doi:10.1038/s41467-021-22817-6)
Supplement: Supplementary file 1 — Supplementary Information [file 41467_2021_22817_MOESM1_ESM.pdf]

SUPPLEMENTARY INFORMATION

TITLE: Differential chromatin binding of the lung lineage transcription factor NKX2-1 resolves opposing murine alveolar cell fates in vivo

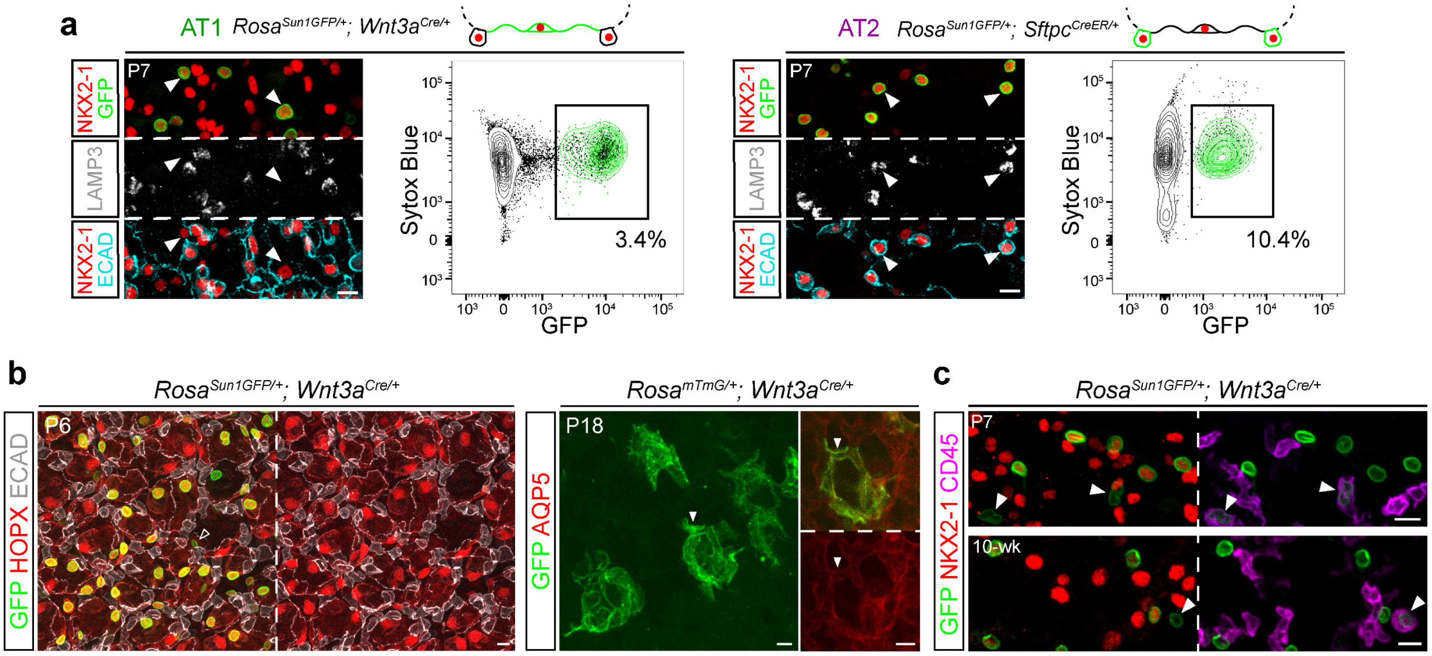

Supplementary Fig. 1: Additional validation of the $Wnt3a^{Cre}$ and $Sftpc^{CreER}$ drivers.

a, Genetic labeling and FACS purification of AT1 and AT2 nuclei at P7 using $Wnt3a^{Cre}$ and $Sftpc^{CreER}$, respectively. The non-AT1 cells targeted by $Wnt3a^{Cre}$ were mostly immune cells (3.3% of GFP+ cells), which did not express NKX2-1, and occasional AT2 cells (0.7% of GFP+ cells). Confocal images of immunostained lungs showing that all alveolar cells express NKX2-1 while AT2 cells are marked by LAMP3 and cuboidal E-Cadherin (ECAD). $Rosa^{Sun1GFP}$ marks the nuclear envelop of recombined cells (arrowheads; green in the diagram). All nuclei are Sytox Blue positive. For $Sftpc^{CreER}$, 250 ug tamoxifen was administrated 3 days before tissue harvest. Scale: 10 µm.

b, Confocal images of immunostained lungs showing that $Wnt3a^{Cre}$ labels AT1 cells marked by HOPX without a cuboidal ECAD outline (left panels) and with extended morphology (arrowhead in right panels). Scale: 10 µm.

c, Confocal images of immunostained lungs showing labeling of immune cells (CD45) by $Wnt3a^{Cre}$, which do not express NKX2-1 and thus do not interfere with NKX2-1 ChIP-seq. Scale: 10 µm.

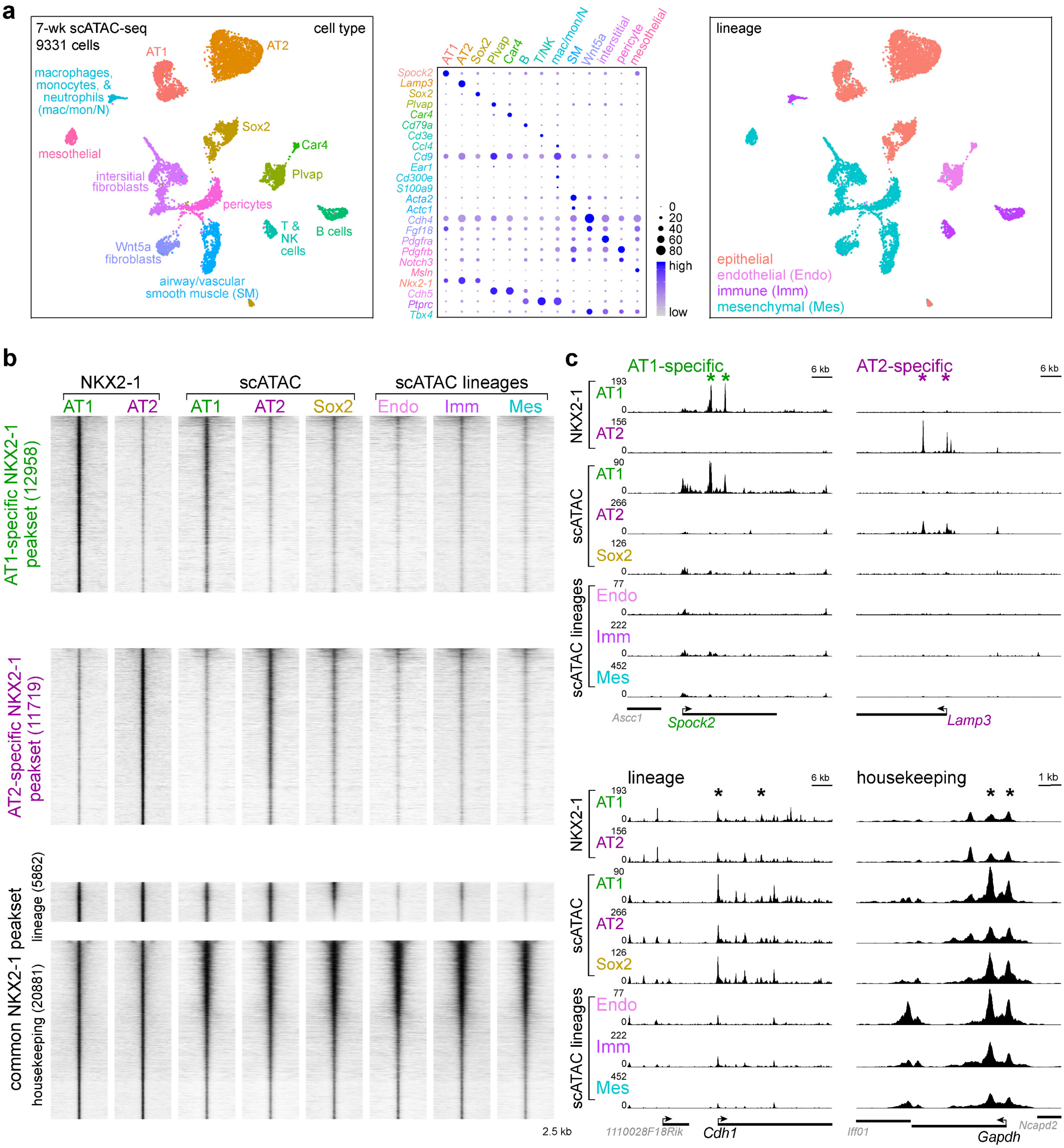

Supplementary Fig. 2: ScATAC-seq deconvolves NKX2-1 ChIP-seq binding into lineage and housekeeping peaksets.

a, ScATAC-seq UMAPs of indicated cell types (abbreviation in parenthesis for the dot plot) and lineages (abbreviation in parenthesis for **b**, **c**), as supported by the corresponding markers in the dot plot.

b, NKX2-1 ChIP-seq heatmaps grouped by differential NKX2-1 binding as in Fig. 1 and cross-referenced to scATAC-seq heatmaps from **a**. Cell-type-specific NKX2-1 binding sites have higher scATAC-seq signals in the

corresponding cell type; NKX2-1 lineage sites have higher scATAC-seq signals in the epithelial cell types; whereas NKX2-1 housekeeping sites have comparable scATAC-seq signals across lineages. NKX2-1 lineage sites are sorted by scATAC-seq signals in Sox2 airway cells to show a subset (bottom) with less accessibility in airway cells than alveolar cells.

c, Example NKX2-1 binding sites and scATAC-seq signals of the AT1-specific (*Spock2*), AT2-specific (*Lamp3*), lineage (*Cdh1*), and housekeeping (*Gapdh*) sets.

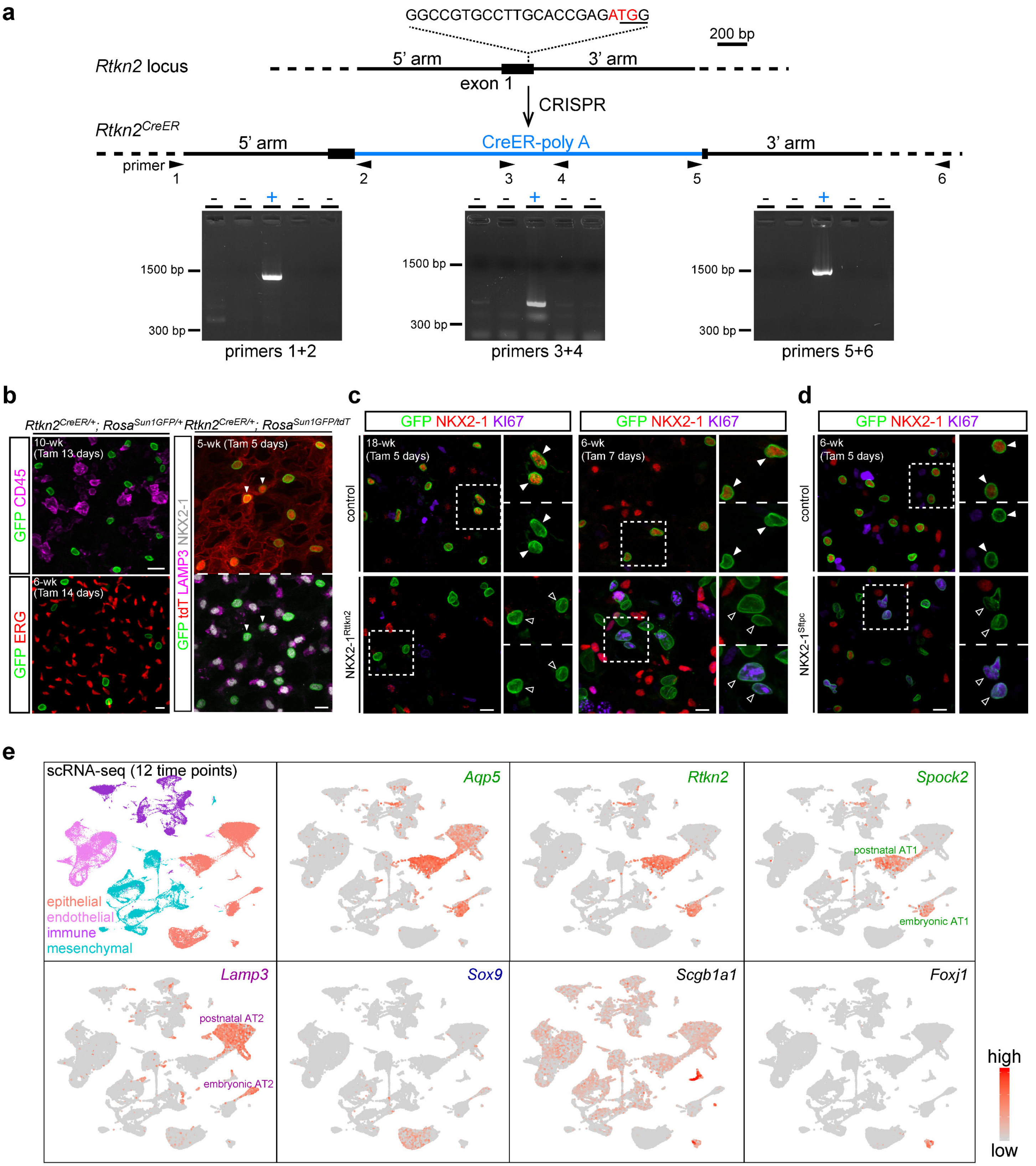

Supplementary Fig. 3: Generation of *Rtkn2*^{CreER} mice and proliferation in *Nkx2-1* mutants.

a, CRISPR (clustered regularly interspaced short palindromic repeats) targeting of the *Rtkn2* locus. The gRNA sequence is shown with the protospacer adjacent motif (PAM; not included in gRNA) underlined; the

translation start of *Rtkn2* (ATG in red) is replaced by that of CreER. Locus-specific PCR genotyping identifies a positive founder mouse. The primer sequences are: #1, 5'-CTCTGTATTCAGTGTAGGGGGGCG; #2, 5'-GTCCATCAGGTTCTTGCGAACCTC; #3, 5'-ATACCGGAGATCATGCAAGC; #4, 5'-AGGTGGACCTGATCATGGAG; #5, 5'-TTCTAGTTGTGGTTTGTCCAAACT; #6, 5'-CTGTGAAGTTGAAACCCATTAACA. See Source Data 3 for uncropped gels.

b, Confocal images of immunostained lungs showing $Rtkn2^{CreER}$ does not target immune (CD45) or endothelial (ERG) cells (left panels), but labels AT1 cells with extended morphology (arrowhead) and without LAMP3 (right panels). Two doses (1.5 mg) of tamoxifen (Tam) were administered for the top left panel and one dose (2 mg) for the bottom left panel and right panels. Scale: 10 μm.

c, **d**, Confocal images of immunostained lungs of $NKX2\text{-}1^{Rtkn2}$ (c) and $NKX2\text{-}1^{Sftpc}$ (d) mutants and their littermate controls, as in Fig. 2, showing ectopic proliferative (KI67) cells 7 and 5 days after tamoxifen (Tam) administration, respectively. Proliferation is rare 5 days after tamoxifen in the $NKX2\text{-}1^{Rtkn2}$ mutant. Scale: 10 μm.

e, UMAPs of the 12 scRNA-seq dataset including all four lineages, showing *Aqp5*, *Rtkn2*, and *Spock2* are specific to AT1 cells. Embryonic and postnatal AT1 or AT2 cells form distinct clusters.

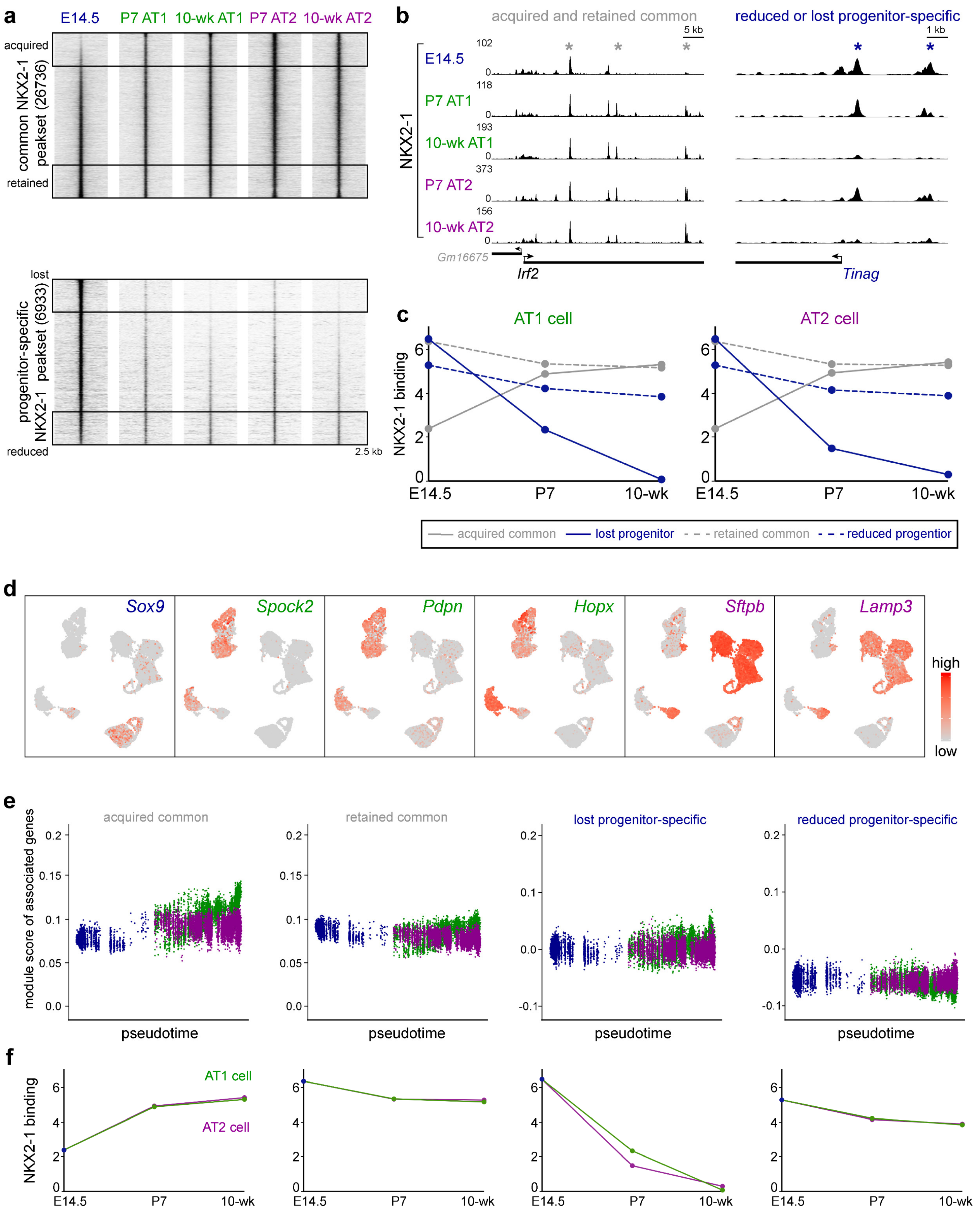

a
E14.5
P7 AT1
10-wk AT1
P7 AT2
10-wk AT2
acquired
common NKX2-1 peakset (26736)
retained
lost
progenitor-specific NKX2-1 peakset (6933)
reduced
2.5 kb
b
acquired and retained common
reduced or lost progenitor-specific
5 kb
1 kb
NKX2-1
E14.5
P7 AT1
10-wk AT1
P7 AT2
10-wk AT2
102
118
193
373
156
Gm16675
Irf2
Tinag
c
AT1 cell
AT2 cell
NKX2-1 binding
E14.5
P7
10-wk
acquired common
lost progenitor
retained common
reduced progentior
d
Sox9
Spock2
Pdpn
Hopx
Sftpb
Lamp3
high
low
e
acquired common
retained common
lost progenitor-specific
reduced progenitor-specific
module score of associated genes
pseudotime
f
AT1 cell
AT2 cell
NKX2-1 binding
E14.5
P7
10-wk

Supplementary Fig. 4: Additional temporal analysis of NKX2-1 binding.

a, NKX2-1 ChIP-seq heatmaps from E14.5 whole lungs, purified AT1 or AT2 nuclei from P7 or 10-week-old lungs, grouped as common (top) or progenitor-specific (bottom) peaksets and sorted by differential NKX2-1 binding between E14.5 lungs and the average of 10-week-old AT1 and AT2 cells. As in Fig. 3 and 4, the top (acquired or lost) and bottom (retained or reduced) 20% sites (boxed area) are used for subsequent analysis. The acquired common sites have little binding in progenitors and increase over time in both AT1 and AT2 cells, while the retained common sites have comparable binding in progenitor, AT1, and AT2 cells. The lost or reduced progenitor-specific sites have high binding in progenitors but decrease drastically or to a lesser extent, respectively, over time in both AT1 and AT2 cells.

b, Example NKX2-1 binding sites (asterisk) of acquired or retained common sites (*Irf2*; both types near the same gene) and lost or reduced sites (*Tinag*).

c, NKX2-1 binding signal of the indicated four categories in **a**, showing the kinetics of common and progenitor-specific sites as AT1 (left) versus AT2 (right) cells mature.

d, ScRNA-seq feature plots validating progenitor (*Sox9*), AT1 (*Spock2*, *Pdpn*, and *Hopx*), and AT2 (*Sftpb* and *Lamp3*) cells in Fig. 4a.

e, Seurat module scores of gene sets associated with acquired or retained common NKX2-1 sites and lost or reduced progenitor-specific sites as defined in **a**, plotted along the Monocle trajectories as in Fig. 4.

f, NKX2-1 binding signal of the four categories as in **e** in AT1 or AT2 cells over time, using data from **c**. Acquired common sites are associated with an increase in the module score over time, albeit more gradually in AT1 cells, while retained common sites have relatively constant module scores. Neither lost nor reduced progenitor-specific sites are associated with a decrease in the module score, possibly due to removal of proliferative cells in the Monocle trajectory analysis.

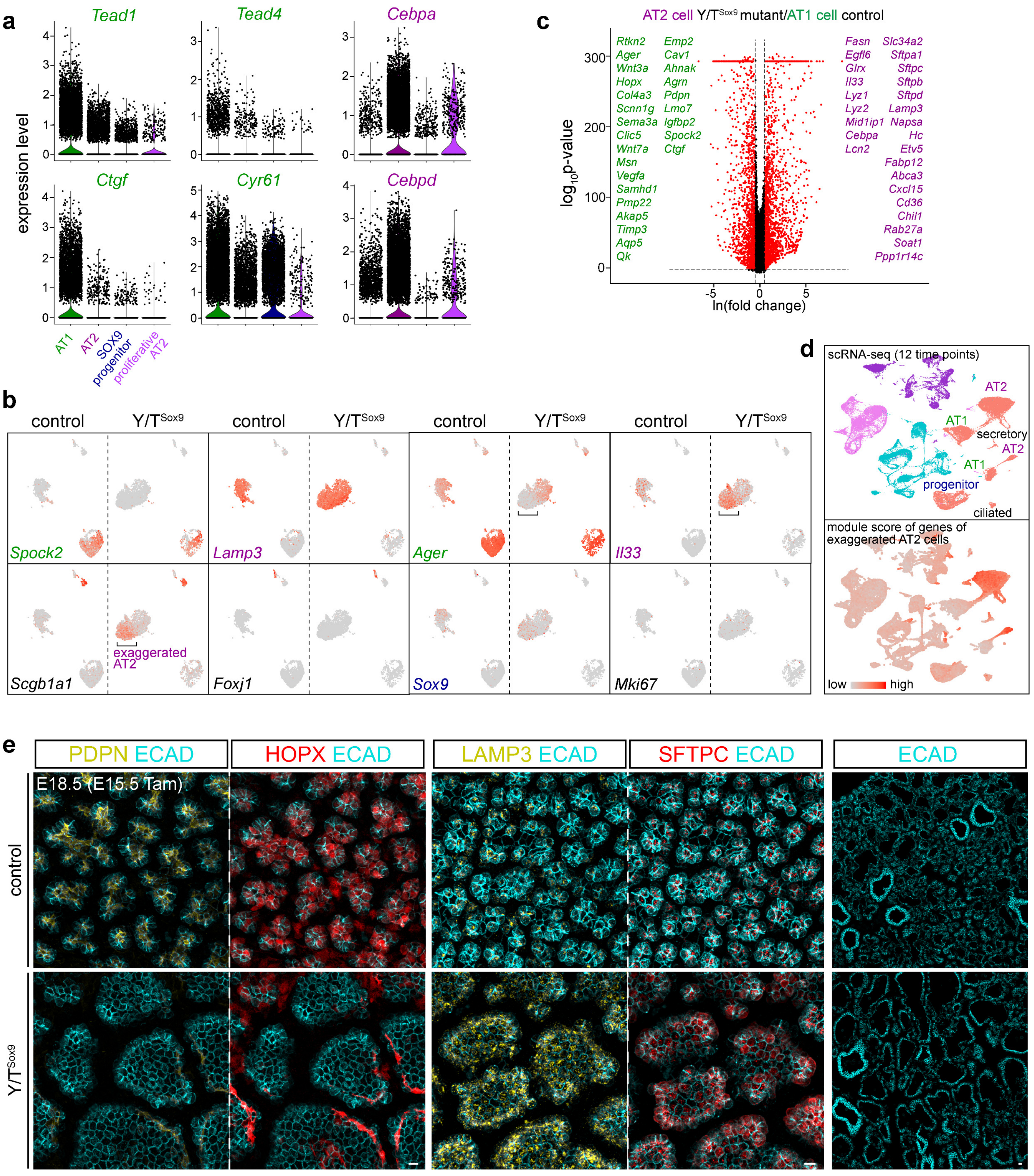

Supplementary Fig. 5: Additional analysis of YAP/TAZ as partner factors for NKX2-1 during development.

a, Violin plots of scRNA-seq data from Fig. 4 in color-coded cell types, showing enrichment of *Tead1/4* and Hippo signaling target genes *Ctgf* and *Cyr61* in AT1 cells and *Cebpa/d* in AT2 cells.

b, Feature plots of scRNA-seq comparison of Y/T^{Sox9} mutant and littermate control lungs, as in Fig. 5. The left half (bracket; exaggerated AT2 cells) of the AT2 cell cluster in the Y/T^{Sox9} mutant loses the low expression of an AT1 gene (*Ager*) but ectopically expresses *Il33* and *Scgb1a1*, the latter of which has a low level of expression in control AT2 cells.

c, Volcano plot of scRNA-seq data from Fig. 5 (MAST differential expression) comparing AT1 cells in the control lung to AT2 cells in the Y/T^{Sox9} mutant, which arise in part from AT1 cells upon *Yap/Taz* deletion, consistent with *Yap/Taz* mutant AT1 cells becoming AT2 cells.

d, Module score analysis of genes associated with exaggerated AT2 cells (the bottom 4 clusters in Fig. 5i) in the 12 scRNA-seq dataset (top panel as in Supplementary Fig. 3e), showing a high score specific to AT2 cells among all major lung cell types (bottom panel).

e, Confocal images of immunostained lungs showing loss of AT1 markers PDPN and HOPX (left panels), wide-spread AT2 markers LAMP3 and SFTPC (middle panels), and air space dilation (right panels) in the Y/T^{Sox9} mutant. Scale: 10 µm.

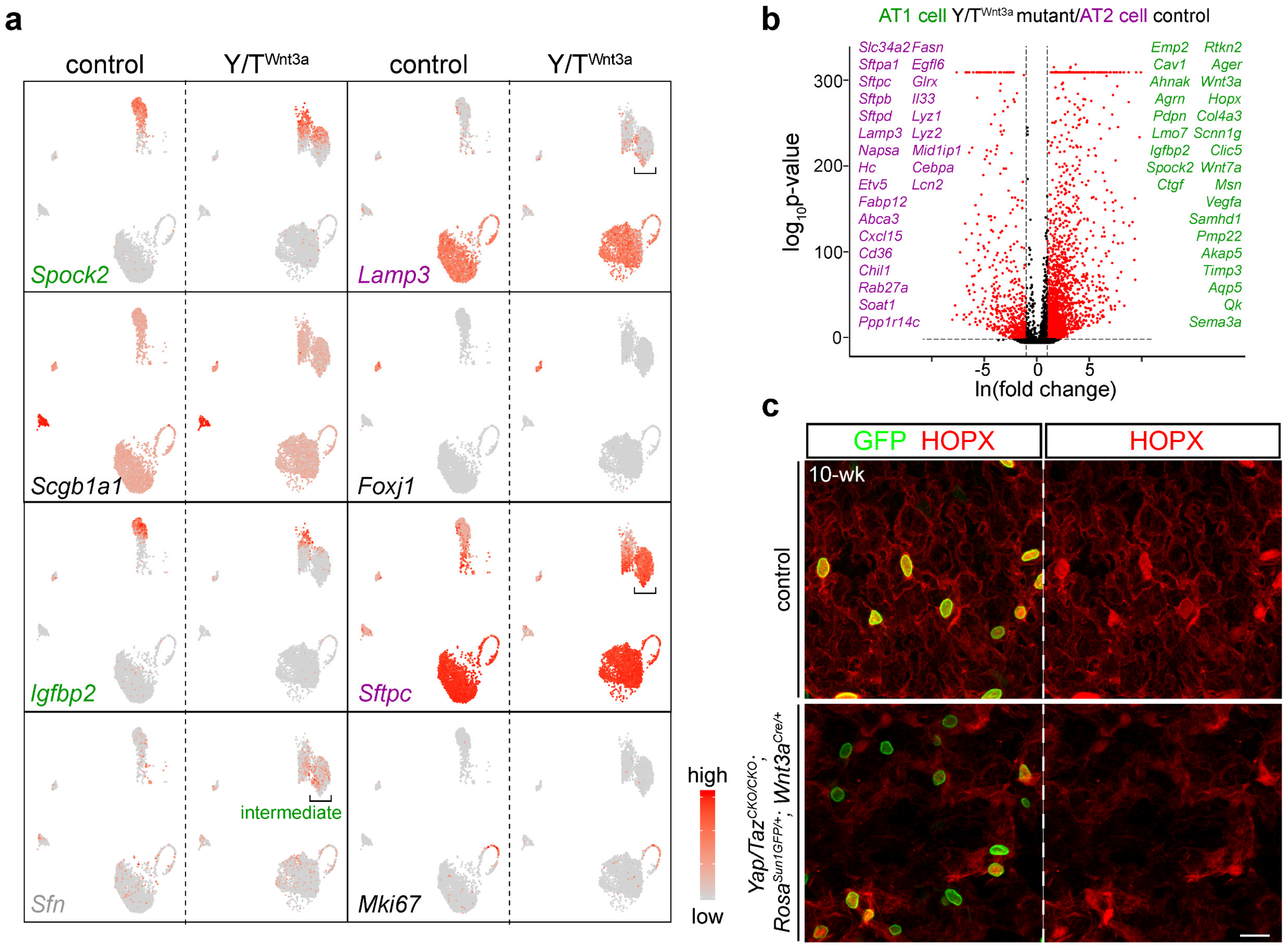

Supplementary Fig. 6: Additional analysis of the Y/T^{Wnt3a} mutant.

a, Feature plots of scRNA-seq comparison of Y/T^{Wnt3a} mutant and littermate control lungs, as in Fig. 6. The right half (bracket; intermediate) of the AT1 cell cluster in the Y/T^{Wnt3a} mutant ectopically expresses AT2 genes *Sftpc* and *Lamp3*, as well as genes implicated in AT2-to-AT1 conversion during injury-repair such as *Sfn*.

b, Volcano plot of scRNA-seq data from Fig. 6 comparing AT2 cells in the control lung to AT1 cells in the Y/T^{Wnt3a} mutant to confirm their expected cell type identity.

c, Confocal images of immunostained littermate lungs showing the loss of an AT1 marker HOPX in the *Yap/Taz* mutant AT1 cells. Some GFP-expressing cells in the mutant lung still express HOPX, likely due to incomplete recombination of all four *Yap/Taz* alleles. Scale: 10 µm.

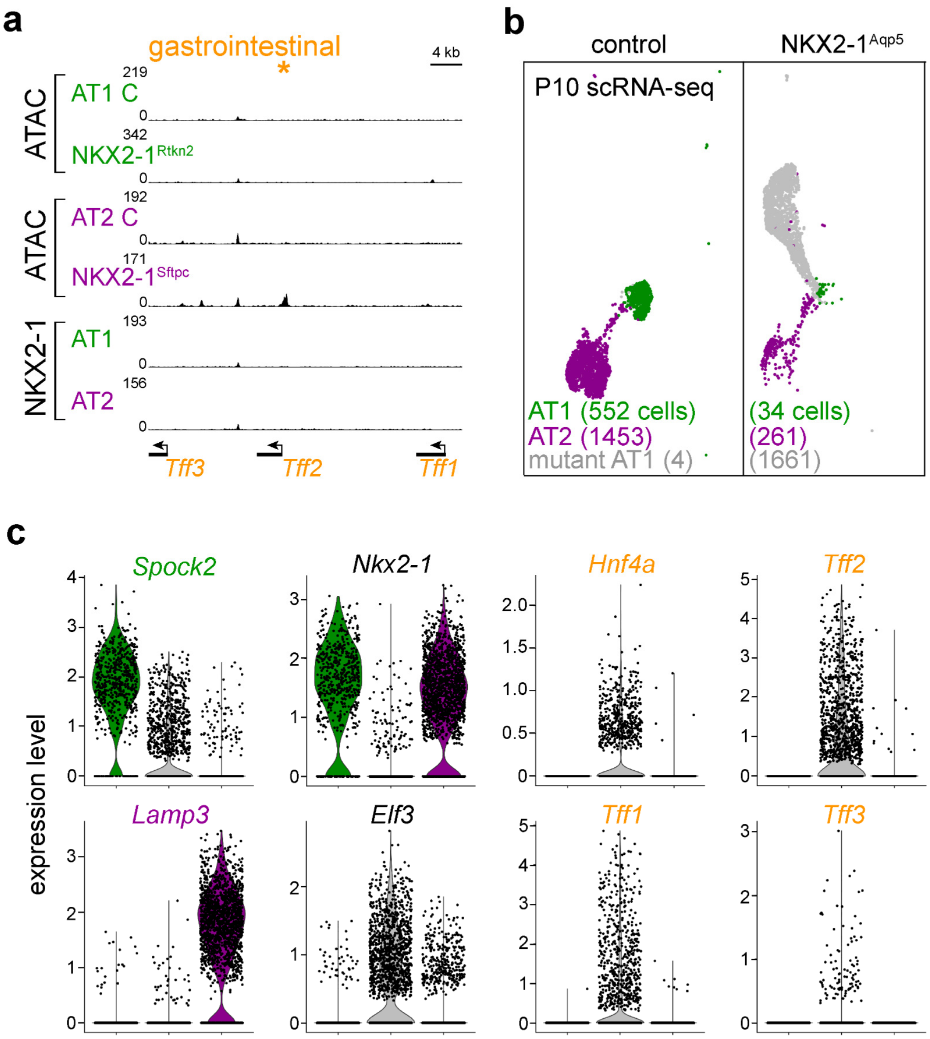

Supplementary Fig. 7: Additional analysis of increased accessibility sites in the *Nkx2-1* mutants.

a, Example gastrointestinal genes (*Tff1/2/3*) with little increase in accessibility except for a small change near *Tff2* in the NKX2-1^{Sftpc} mutant (asterisk), possibly due to faster adoption of the gastrointestinal fate by AT2 cells than AT1 cells.

b, ScRNA-seq UMAPs of our published NKX2-1^{Aqp5} mutant showing the indicated cell types and numbers.

c, Violin plots of the scRNA-seq data in **b**, showing increased expression of gastrointestinal genes including *Hnf4a* and *Tff1/2/3* and a transcription factor with the ELF motif (*Elf3*) in *Nkx2-1* mutant AT1 cells.
