# Supplementary material for: Differential chromatin binding of the lung lineage transcription factor NKX2-1 resolves opposing murine alveolar cell fates in vivo
Source: Nat Commun. 2021 May 4;12:2509. doi: 10.1038/s41467-021-22817-6 (PMC8096971; doi:10.1038/s41467-021-22817-6)
Supplement: Supplementary file 2 — Description of Additional Supplementary Files [file 41467_2021_22817_MOESM2_ESM.pdf]

Title: Supplementary Data 1:
Description: Peaksets for NKX2-1 ChIP-seq heatmaps in Fig. 1, 2.

Title: Supplementary Data 2:
Description: Peaksets for NKX2-1 ChIP-seq heatmaps in Fig. 3.

Title: Supplementary Data 3:
Description: NKX2-1 ChIP-seq peaksets for acquired or retained AT1/AT2-specific and common categories and lost or reduced progenitor-specific categories.

Title: Supplementary Data 4:
Description: Peaksets for NKX2-1 ChIP-seq heatmaps in Fig. 5 as well as locations of TEAD and NKX motifs.

Title: Supplementary Data 5:
Description: Curated AT1 and AT2 genes and scRNA-seq analysis in Fig. 5 (MAST differential expression).

Title: Supplementary Data 6:
Description: Peaksets for NKX2-1 ChIP-seq heatmaps in Fig. 6.

Title: Supplementary Data 7:
Description: Curated AT1 and AT2 genes and scRNA-seq analysis in Fig. 6 (MAST differential expression).

Title: Supplementary Data 8:
Description: Peaksets for NKX2-1^{Rtkn2} ATAC-seq heatmaps in Fig. 7.

Title: Supplementary Data 9:
Description: Peaksets for NKX2-1^{Sftpc} ATAC-seq heatmaps in Fig. 7.

Title: Supplementary Data 10:
Description: MA plot values for Fig. 7.

Title: Supplementary Software File 1:
Description: Custom script used to generate the figures.
