# Supplementary material for: Differential chromatin binding of the lung lineage transcription factor NKX2-1 resolves opposing murine alveolar cell fates in vivo
Source: Nat Commun. 2021 May 4;12:2509. doi: 10.1038/s41467-021-22817-6 (PMC8096971; doi:10.1038/s41467-021-22817-6)
Supplement: Supplementary file 15 — Reporting Summary [file 41467_2021_22817_MOESM15_ESM.pdf]

nature research

Corresponding author(s): Jichao Chen

Last updated by author(s): Mar 12, 2021

Reporting Summary

Nature Research wishes to improve the reproducibility of the work that we publish. This form provides structure for consistency and transparency in reporting. For further information on Nature Research policies, see our Editorial Policies and the Editorial Policy Checklist.

Statistics

For all statistical analyses, confirm that the following items are present in the figure legend, table legend, main text, or Methods section.

n/a	Confirmed	
☐	☒	The exact sample size (*n*) for each experimental group/condition, given as a discrete number and unit of measurement
☐	☒	A statement on whether measurements were taken from distinct samples or whether the same sample was measured repeatedly
☐	☒	The statistical test(s) used AND whether they are one- or two-sided<br>*Only common tests should be described solely by name; describe more complex techniques in the Methods section.*
☒	☐	A description of all covariates tested
☐	☒	A description of any assumptions or corrections, such as tests of normality and adjustment for multiple comparisons
☒	☐	A full description of the statistical parameters including central tendency (e.g. means) or other basic estimates (e.g. regression coefficient) AND variation (e.g. standard deviation) or associated estimates of uncertainty (e.g. confidence intervals)
☐	☒	For null hypothesis testing, the test statistic (e.g. *F*, *t*, *r*) with confidence intervals, effect sizes, degrees of freedom and *P* value noted<br>*Give P values as exact values whenever suitable.*
☒	☐	For Bayesian analysis, information on the choice of priors and Markov chain Monte Carlo settings
☒	☐	For hierarchical and complex designs, identification of the appropriate level for tests and full reporting of outcomes
☒	☐	Estimates of effect sizes (e.g. Cohen's *d*, Pearson's *r*), indicating how they were calculated

Our web collection on statistics for biologists contains articles on many of the points above.

Software and code

Policy information about availability of computer code

Data collection	ChIP-seq were analyzed using using Fastqc, Trimmomatic (0.33), Bowtie (1.2.2), Picard (2.9.0), Samtools(1.10), MACS2, Homer, and Diffbind (2.14.0).<br>ATAC-seq were analyzed using BCL2FASTq, Fastqc, Trimmomatic (0.33), Bowtie2(2.3.4.2), Picard (2.9.0), Samtools(1.10), MACS2, and Diffbind (2.14.0).<br>scRNA-seq were analyzed using cellranger (3.0.2) and the R packages: Seurat (3.1.4.), Monocle (2.14.0)<br>scATAC-seq were analyzed using cellranger-atac (1.2), Sinto (3.7.3), Picard (2.9.0) Samtools(1.10), MACS2, and Diffbind (2.14.0) and R packages: Seurat (3.1.4), Signac (0.1.6),<br>Cell sorting was analyzed using FlowJo (v9)
Data analysis	Custom code for analysis are provided in Supplemental Software File 1 that provides the R script and codes run for analysis so that others can reproduce our analysis.

For manuscripts utilizing custom algorithms or software that are central to the research but not yet described in published literature, software must be made available to editors and reviewers. We strongly encourage code deposition in a community repository (e.g. GitHub). See the Nature Research guidelines for submitting code & software for further information.

Data

Policy information about availability of data

All manuscripts must include a data availability statement. This statement should provide the following information, where applicable:

- Accession codes, unique identifiers, or web links for publicly available datasets
- A list of figures that have associated raw data
- A description of any restrictions on data availability

ChIP-seq, ATAC-seq, scRNA-seq, and scATAC-seq have been deposited at NCBI Gene Expression Omnibus (GEO) under the accession number GSE158205.

Field-specific reporting

Please select the one below that is the best fit for your research. If you are not sure, read the appropriate sections before making your selection.

- [x] Life sciences
- [ ] Behavioural & social sciences
- [ ] Ecological, evolutionary & environmental sciences

For a reference copy of the document with all sections, see nature.com/documents/nr-reporting-summary-flat.pdf

Life sciences study design

All studies must disclose on these points even when the disclosure is negative.

Sample size	For ChIP-seq and ATAC-seq two biological replicates is the standard in the field, while for single-cell data it is acceptable to perform it one time on thousands of cells using 10X Genomics.<br>For phenotype analysis by imaging, no power analysis for sample size was carried out, but our assays rely upon cellular readouts of hundreds to thousands of cells per mouse and 2-3 mice per experimental group, which is sufficient to ensure reproducible results based on prior experience.
Data exclusions	Data was only excluded if technical errors during the experiment were detected.
Replication	All confocal images are representative of at least 3 imaging fields of each sample and at least 3 sets of control and mutant lungs except for Fig. 6h and Supplementary Fig. 6c where 3 control lungs and 2 mutant lungs were used. Hundreds to thousands of cells were quantified in each comparison.<br>ChIP-seq and bulk ATAC-seq experiments was carried out twice using biological replicates.<br>ScRNA-seq and scATAC-seq experiments were carried out once for each condition.
Randomization	Animals were genotyped to identify control and mutant mice. No randomization is needed.
Blinding	Blinding was not used because the complexity in the genetics and the obvious molecular phenotypes.

Reporting for specific materials, systems and methods

We require information from authors about some types of materials, experimental systems and methods used in many studies. Here, indicate whether each material, system or method listed is relevant to your study. If you are not sure if a list item applies to your research, read the appropriate section before selecting a response.

Materials & experimental systems

n/a	Involved in the study	
[ ]	[x]	Antibodies
[x]	[ ]	Eukaryotic cell lines
[x]	[ ]	Palaeontology and archaeology
[ ]	[x]	Animals and other organisms
[x]	[ ]	Human research participants
[x]	[ ]	Clinical data
[x]	[ ]	Dual use research of concern

Methods

n/a	Involved in the study	
[ ]	[x]	ChIP-seq
[ ]	[x]	Flow cytometry
[x]	[ ]	MRI-based neuroimaging

Antibodies

Antibodies used	The following antibodies were used for immunofluorescence: rat anti-protein tyrosine phosphatase, receptor type, C (CD45, 1:2000, 14-0451-81, eBioscience) rabbit anti-CCAAT/enhancer binding protein alpha (C/EBPA, 1:500, 8178P, Cell Signaling Technology), rat anti-epithelial cadherin (ECAD, 1:1000, 13190, Life Technology), chicken anti-green fluorescent protein (GFP, 1:5000, AB13970, Abcam), rabbit anti-homeodomain only protein (HOPX, 1:500, sc-30216, Santa Cruz), rat anti-KI67 (KI67, 1:1000, 14-5698-82,

Invitrogen), guinea pig anti-lysosomal associated membrane protein 3 (LAMP3, 1:500, 391005, SySy), rabbit anti-NK homeobox 2-1 (NKX2-1, 1:1000, sc-13040, Santa Cruz), goat anti-podoplanin (PDPN, 1:1000, AF3244, R&D), goat anti-polymeric immunoglobulin receptor (PIGR, 1:1000, AF2800, R&D), rabbit anti-pro-surfactant protein C (SFTPC, 1:1000, AB3786, Millipore), rabbit anti-trefoil factor 2 (TFF2, 1:1000, 13681-1-AP, ProteinTech), rabbit anti-Yes-associated protein 1 and WW domain containing transcription regulator 1 (YAP1 and WWTR1/TAZ, 1:250, D24E4, Cell Signaling Technology). The following antibodies were used for fluorescence activated cell sorting: PE/Cy7 rat anti-CD45 (CD45, 1:250, 103114, BioLegend), PE rat anti-epithelial cadherin (ECAD, 1:250, 147304, BioLegend), BV421 rat anti-epithelial cell adhesion molecule (EPCAM, 1:250, 118225, BioLegend), and AF647 rat anti-Intercellular adhesion molecule 2 (ICAM2, 1:250, A15452, ThermoFisher).

The following antibodies were used for chromatin immunoprecipitation: rabbit anti-histone H3 lysine 27 acetylation (H3K27ac, 1 µg/ml, ab4729, Abcam), rabbit anti-Histone H3 lysine 4 mono-methylation (H3K4me1, 0.6 µg/ml, ab8895, Abcam), rabbit anti-Histone H3 lysine 4 tri-methylation (H3K4me3, 1 µg/ml, ab8580, Abcam), and rabbit anti-NK Homeobox 2-1 (NKX2-1, 1 µg/ml, ab133737, Abcam).

Validation

Antibodies used in our study were validated as noted by their suppliers and additionally verified in our hands by their predicted subcellular localization and cell-type specificity based on RNA expression. ChIP-seq histone antibodies were further validated by the ENCODE database. NKX2-1 antibodies were validated by immunostaining in Nkx2-1 knockout mice and multiple NKX2-1 antibodies produced consistent ChIP-seq results as demonstrated in Little et al, 2019, PNAS.

Animals and other organisms

Policy information about studies involving animals; ARRIVE guidelines recommended for reporting animal research

Laboratory animals

All mouse strains used in this study were maintained in mixed genetic backgrounds. Whenever possible, littermate control and mutant mice were used. All experiments conducted were conducted on mixed populations of male and female. The animals were housed at 22oC, 45% humidity, and 12-12 hour light-dark cycle conditions. The ages described in the manuscript are as follows: RosaSun1GFP/+; SftpcCreER/+ were sampled at P7 and 10-wk. RosaSun1Gfp/+;Wnt3aCre/+ were sampled at P7, P15, and 10-wk.. RosaSun1GFP/+; Nkx2-1CKO/CKO; Rtkn2CreER were collected at 6-wk, 10-wk, RosaSun1GFP/+; Nkx2-1 CKO/CKO; SftpcCreER were collected at 6-wk and 17-wk, Yap/Taz CKO/CKO; Sox9CreER/+ mice and littermate controls were collected at E18.5. RosaSun1GFP/+; Yap/Taz CKO/CKO; Wnt3aCre/+ mice were collected at P15 and 10-wk.

Wild animals

No wild animals were used in this study.

Field-collected samples

No field collected samples were used in this study.

Ethics oversight

All animal experiments were approved by the Institutional Animal Care and Use Committee at MD Anderson Cancer Center

Note that full information on the approval of the study protocol must also be provided in the manuscript.

ChIP-seq

Data deposition

☒ Confirm that both raw and final processed data have been deposited in a public database such as GEO.

☒ Confirm that you have deposited or provided access to graph files (e.g. BED files) for the called peaks.

Data access links
May remain private before publication.

GSE158205

Files in database submission

ChIP-seq replicates and respective inputs, ATAC-seq, scRNA-seq, and scATAC-seq

Genome browser session
(e.g. UCSC)

not needed.

Methodology

Replicates

At least two biological replicates were used.

Sequencing depth

At least 10 million uniquely mapped, high quality, non duplicated, 75bp reads (single-end for ChIP-seq and paired-end for ATAC-seq) were used.

Antibodies

The following antibodies were used for chromatin immunoprecipitation: rabbit anti-histone H3 lysine 27 acetylation (H3K27ac, 1 µg/ml, ab4729, Abcam), rabbit anti-Histone H3 lysine 4 mono-methylation (H3K4me1, 0.6 µg/ml, ab8895, Abcam), rabbit anti-Histone H3 lysine 4 tri-methylation (H3K4me3, 1 µg/ml, ab8580, Abcam), and rabbit anti-NK Homeobox 2-1 (NKX2-1, 1 µg/ml, ab133737, Abcam).

Peak calling parameters

Fastqc: fastqc -f fasta -t 8 sample.fastq

Trimmomatic: java -jar $TRIMMOMATIC/trimmomatic-0.33.jar SE -phred33 -threads 6 sample.fastq SLIDINGWINDOW:4:15 MINLEN:21

Bowtie: bowtie -p 24 -k 1 -v 1 -m 1 -S ~/mm10.bt sample.fastq

Samtools:
1. sort : samtools view -bS sample.sam > sample.s.bam

Picard: java -Xmx8g -jar ~/picard-2.18.27/picard.jar MarkDuplicates I=sample.s.bam ASSUME_SORTED=true METRICS_FILE=sample.s.markdup.metrics VALIDATION_STRINGENCY=LENIENT OUTPUT=sample.s.md.bam

Samtools:
2. index: samtools index sample.s.md.bam
3. filter: samtools view -b -h -F 4 -F 1024 -F 2048 -q 30 sample.s.md.bam > sample.sf.md.bam

MACS2:
Sharp: macs2 callpeak -t sample.sf.md.bam -c input.sample.sf.md.bam -g mm -n -B -f BAM
Broad: macs2 callpeak -t sample.sf.md.bam -c input.sample.sf.md.bam -broad -g mm -n -B -f BAM

Information about individual replicates and their paired inputs are available at GEO158205.

Data quality

We followed the ENCODE standard and recommended software settings for data processing, and visually verified at least 20 identified peaks in each sample. The numbers of peaks in each sample are listed below.
GEO name Sample name FDR 5% total peaks FDR 5% logfc 5 peaks
GSM4795138 E14-5 whole lung NKX2-1 ChIP-seq rep 1 64,000 38,000
GSM4795139 E14-5 whole lung NKX2-1 ChIP-seq rep 2 41,000 27,000
GSM4795140 E18-5 whole lung NKX2-1 ChIP-seq rep 1 85,000 55,000
GSM4795141 E18-5 whole lung NKX2-1 ChIP-seq rep 2 88,000 57,000
GSM4795142 E18-5 YT Sox9 CreER whole lung NKX2-1 ChIP-seq rep 1 96,000 64,000
GSM4795143 E18-5 YT Sox9 CreER whole lung NKX2-1 ChIP-seq rep 2 108,000 70,000
GSM4795144 P7 AT1 cell NKX2-1 ChIP-seq rep 1 63,000 48,000
GSM4795145 P7 AT1 cell NKX2-1 ChIP-seq rep 2 75,000 61,000
GSM4795146 P15 AT1 cell NKX2-1 ChIP-seq rep 1 83,000 59,000
GSM4795147 P15 AT1 cell NKX2-1 ChIP-seq rep 2 54,000 83,000
GSM4795148 P15 YT Wnt3a AT1 cell NKX2-1 ChIP-seq rep 1 65,000 47,000
GSM4795149 P15 YT Wnt3a AT1 cell NKX2-1 ChIP-seq rep 2 27,000 53,000
GSM4795150 10wk AT1 cell NKX2-1 ChIP-seq rep 1 89,000 63,000
GSM4795151 10wk AT1 cell NKX2-1 ChIP-seq rep 2 80,000 60,000
GSM4795152 P7 AT2 cell NKX2-1 ChIP-seq rep 1 109,000 80,000
GSM4795153 P7 AT2 cell NKX2-1 ChIP-seq rep 2 111,000 78,000
GSM4795154 10wk AT2 cell NKX2-1 ChIP-seq rep 1 80,000 62,000
GSM4795155 10wk AT2 cell NKX2-1 ChIP-seq rep 2 72,000 55,000
GSM4795156 P10 whole lung H3K4me3 ChIP-seq 23,000 19,000
GSM4795157 P7 AT1 cell H3K4me3 ChIP-seq rep 1 33,000 27,000
GSM4795158 P7 AT1 cell H3K4me3 ChIP-seq rep 2 29,000 24,000
GSM4795159 P7 AT2 cell H3K4me3 ChIP-seq rep 1 40,000 27,000
GSM4795160 P7 AT2 cell H3K4me3 ChIP-seq rep 2 35,000 27,000
GSM4795161 10wk AT1 cell H3K4me3 ChIP-seq rep 1 37,000 26,000
GSM4795162 10wk AT1 cell H3K4me3 ChIP-seq rep 2 39,000 26,000
GSM4795163 10wk AT1 cell H3K27ac ChIP-seq rep 1 85,000 39,000
GSM4795164 10wk AT1 cell H3K27ac ChIP-seq rep 2 107,000 39,000
GSM4795165 10wk AT1 cell H3K4me1 ChIP-seq rep 1 123,000 5,200
GSM4795166 10wk AT1 cell H3K4me1 ChIP-seq rep 2 141,000 3,600
GSM4795167 10wk AT2 cell H3K4me3 ChIP-seq rep 1 42,000 31,000
GSM4795168 10wk AT2 cell H3K4me3 ChIP-seq rep 2 45,000 30,000
GSM4795169 10wk AT2 cell H3K27ac ChIP-seq rep 1 114,000 47,000
GSM4795170 10wk AT2 cell H3K27ac ChIP-seq rep 2 110,000 50,000
GSM4795171 10wk AT2 cell H3K4me1 ChIP-seq rep 1 130,000 1,700
GSM4795172 10wk AT2 cell H3K4me1 ChIP-seq rep 2 120,000 600

Software

ChIP-seq were analyzed using using Fastqc, Trimmomatic (0.33), Bowtie (1.2.2), Picard (2.9.0), Samtools (1.10), MACS2, Homer, and Diffbind (2.14.0). Custom script is provided in the Supplementary Software File 1 and individual commands are also references in the methods section of this study.

Flow Cytometry

Plots

Confirm that:

- [x] The axis labels state the marker and fluorochrome used (e.g. CD4-FITC).
- [x] The axis scales are clearly visible. Include numbers along axes only for bottom left plot of group (a 'group' is an analysis of identical markers).
- [x] All plots are contour plots with outliers or pseudocolor plots.
- [x] A numerical value for number of cells or percentage (with statistics) is provided.

Methodology

Sample preparation	Lungs were harvested from Avertin anesthetized mice after perfusing 3 mL of cold PBS through the right ventricle. Lungs were minced after removal of extra-pulmonary tissues. The tissue was then crosslinked for 20 min on a rocker at room temperature using a 1:4 PBS diluted 10% buffered formalin (ThermoFisher Scientific, 23-245-685). To quench the excess fixative, 1 M glycine (pH 5.0) was added to a final concentration of 125 mM and incubated at room temperature on a rocker for 5-10 min. The fixed tissue was then washed with 2 mL cold PBS and resuspended with 1 mL (500 µL for embryos) of isolation of nuclei tagged in specific cell types (INTACT16) buffer (20 mM HEPES pH 7.4, 25 mM KCl, 0.5 mM MgCl2, 0.25 M sucrose, 1 mM DTT, 0.4% NP-40, 0.5 mM Spermine, 0.5 mM Spermidine) with protease inhibitor cocktail (cOmplete ULTRA Tablets, Mini, EDTA-free, EASY pack, Sigma, 5892791001 or Pierce Protease Inhibitor Mini Tablets, EDTA-free, ThermoFisher Scientific, A32955). Resuspended samples were then Dounce homogenized for 5 strokes, filtered through a 70 µm cell strainer, and centrifuged in a 2 mL tube at 384 rcf for 5 min. Samples were then resuspended in PBS plus protease inhibitor cocktail and either sorted for cell-type-specific ChIP-seq or counted for whole lung ChIP-seq. For cell-type-specific ChIP-seq, nuclei were stained with Sytox blue (1:1000, Invitrogen, S34857) then filtered through a 35m cell strainer into a 5 mL glass tube (12x75mm Culture Tubes with closures volume 5 mL, VWR, 60818-565) blocked with 200 µL 10 mg/mL BSA (Sigma, A3059) and 1x protease inhibitor cocktail. Nuclei were then sorted at 4°C using a BD FACSAria Fusion sorter or BD FACSAria IIIu cell sorter for GFP+ nuclei from the RosaSun1GFP allele and Sytox blue positive nuclei into a 1.7 ml collection tube containing and blocked with 300 µL of 10 mg/mL BSA with 5x protease inhibitor cocktail. Wnt3aCre/+; RosaSun1GFP/+ mice rendered ~1 million nuclei per lung. SftpcCreER/+; RosaSun1GFP/+ mice rendered 1-2 million nuclei per lung. For a full set of histones ChIPs in addition to an NKX2-1 ChIP, nuclei from lungs of mice with the same genotype were combined. If samples were combined, a second experiment with different mice of the same genotype and time point would be performed for a biological replicate.
Instrument	Both cells and nuclei were sorted in either the BD FACSAria Fusion sorter or BD FACSAria IIIu cell sorter.
Software	FlowJo (v9)
Cell population abundance	Wnt3aCre GFP labeled nuclei ranged from 1-5% of the total nuclei while SftpcCreER GFP labeled nuclei comprised 5-11% of the nuclei. The percentages are consistent with immunostaining on sections (Fig. 1a). ChIP-seq using sorted nuclei also shows the expected enrichment for cell-type-specific genes (Fig. 1b).<br><br>Rtkn2CreER GFP labeled cells ranged from 1-3%, while SftpcCreER GFP labeled cells comprised 1-2% of the live cell population. Cell sorting is less efficient than nucleus sorting because epithelial cells are preferentially lost during cell dissociation. The percentages are consistent with immunostaining on sections (Fig. 2a and 2b). ChIP-seq using sorted nuclei also shows the expected enrichment for cell-type-specific genes (Fig. 2c and 2d).<br><br>For single cell experiments, CD45+ cells comprised ~30% of live cells, mesenchymal cells were ~20%, endothelial cells were 40% while epithelial cells were 10% of total live cells. The precentages are consistent with the populations identified in scRNA-seq and scATAC-seq results (sup. Fig. 2b and sup. Fig. 3e).
Gating strategy	All sorts regardless of nuclei or cells started with gating of cells based on SSC-A vs FSC-A to get ride of small debris. The following gate for all sorts was FSC-A and FSC-H to get rid of doublets. When necessary, a second round of gating with SSC-A and SSC-H was used to further remove doublets. Nuclei were gated for positive SYTOX blue while live cells negative. GFP nuclei or cells were gated on FITC-A. A histogram helped determine a cutoff.<br><br>For single cell experiments, after selection of live cells using SYTOX blue, CD45-PE-Cyt7-A was use to separate CD45+ immune cells from CD45- cells. The boundary between these populations was based on a contour plot depicting a gap between the two populations. All boundaries between populations were minimized to maximize capture of every cell type in the lung. The CD45- population was then separated again by a contour plot using ICAM2-A647 APC-A to decide ICAM2 positive endothelial cells versus non-endothelial non-immune cells. CD45- ICAM2- cells were then separated based on ECAD using a contour plot to determine the boundary between epithelial and mesenchymal cells.

- [x] Tick this box to confirm that a figure exemplifying the gating strategy is provided in the Supplementary Information.
