# Supplementary material for: Differential chromatin binding of the lung lineage transcription factor NKX2-1 resolves opposing murine alveolar cell fates in vivo
Source: Nat Commun. 2021 May 4;12:2509. doi: 10.1038/s41467-021-22817-6 (PMC8096971; doi:10.1038/s41467-021-22817-6)
Supplement: Supplementary file 16 — Source Data [file 41467_2021_22817_MOESM16_ESM.zip › Source Data 2.pdf]

Source Data 2: Sorting schemes for flow cytometry data.

1. Sorting scheme for collecting GFP positive nuclei from P7, P15, and 10-wk *Rosa*$^{Sun1GFP/+}$*;* *Wnt3a*$^{Cre/+}$ mouse lungs and for P15 *Yap/Taz*$^{CKO/CKO}$*; Rosa*$^{Sun1GFP/+}$*; Wnt3a*$^{Cre/+}$. The example below is of sorting GFP positive cells from 10-wk *Rosa*$^{Sun1GFP/+}$*; Wnt3a*$^{Cre/+}$ mouse lungs.

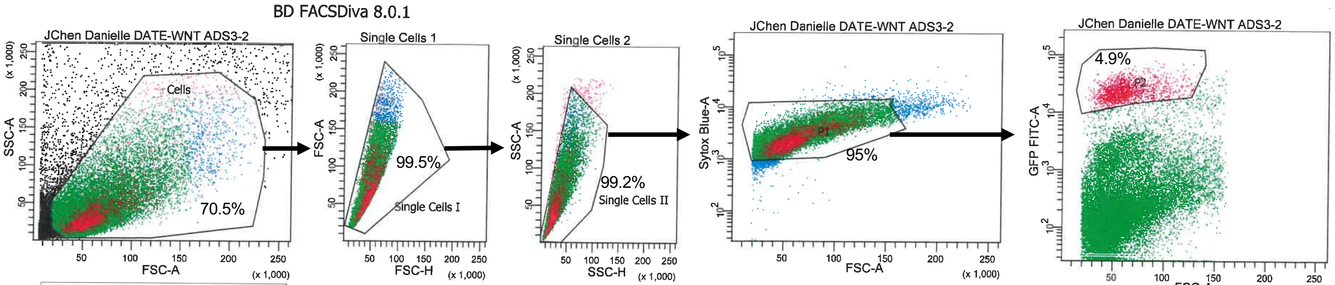

Tube: WNT ADS3-2

Population	#Events	%Parent	%Total
All Events	50,000	####	100.0
Cells	35,264	70.5	70.5
Single Cells I	35,091	99.5	70.2
Single Cells II	34,798	99.2	69.6
P1	33,053	95.0	66.1
P2	1,613	4.9	3.2

2. Sorting scheme for collecting GFP positive nuclei from P7 and 10-wk Rosa$^{Sun1GFP/+}$; Sftpc$^{CreER/+}$ mouse lungs. The example below is of sorting GFP positive cells from 10-wk *Rosa*$^{Sun1GFP/+}$*; Sftpc*$^{CreER/+}$ mouse lungs.

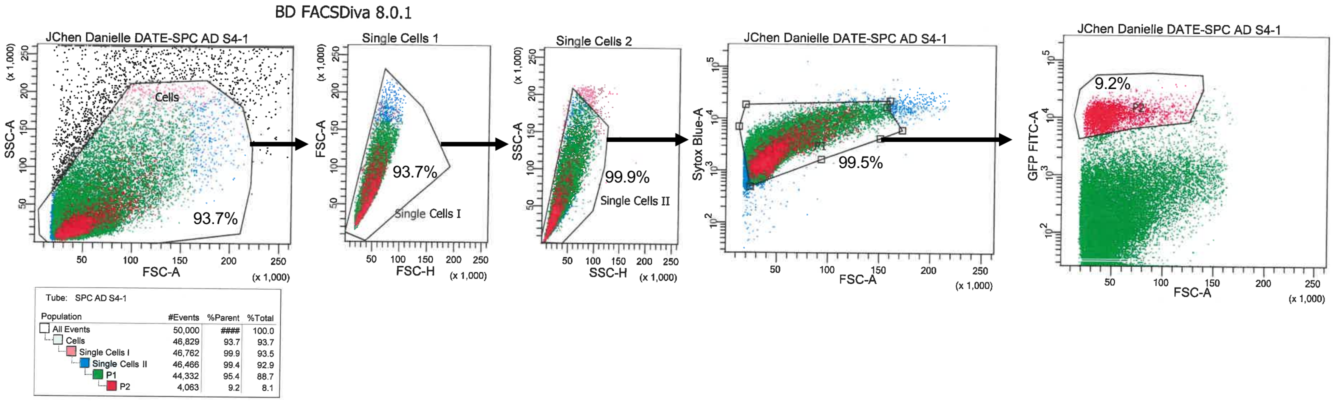

Tube: SPC AD S4-1

Population	#Events	%Parent	%Total
All Events	50,000	####	100.0
Cells	46,829	93.7	93.7
Single Cells I	46,762	99.9	93.5
Single Cells II	46,466	99.4	92.9
P1	44,332	95.4	88.7
P2	4,063	9.2	8.1

3. Sorting scheme for collecting immune, endothelial, epithelial, and mesenchymal populations for E14.5, E16.5, E18.5, E18.5 *Yap/Taz*$^{CKO/CKO}$; *Sox9*$^{CreER/+}$, P4, P6, P7, P8, P10, P20, 10-wk, and 15-wk mouse lungs for scRNA-seq and 10-wk mouse lungs for scATAC-seq. The example below is of sorting 10-wk mouse lungs for scATAC-seq.

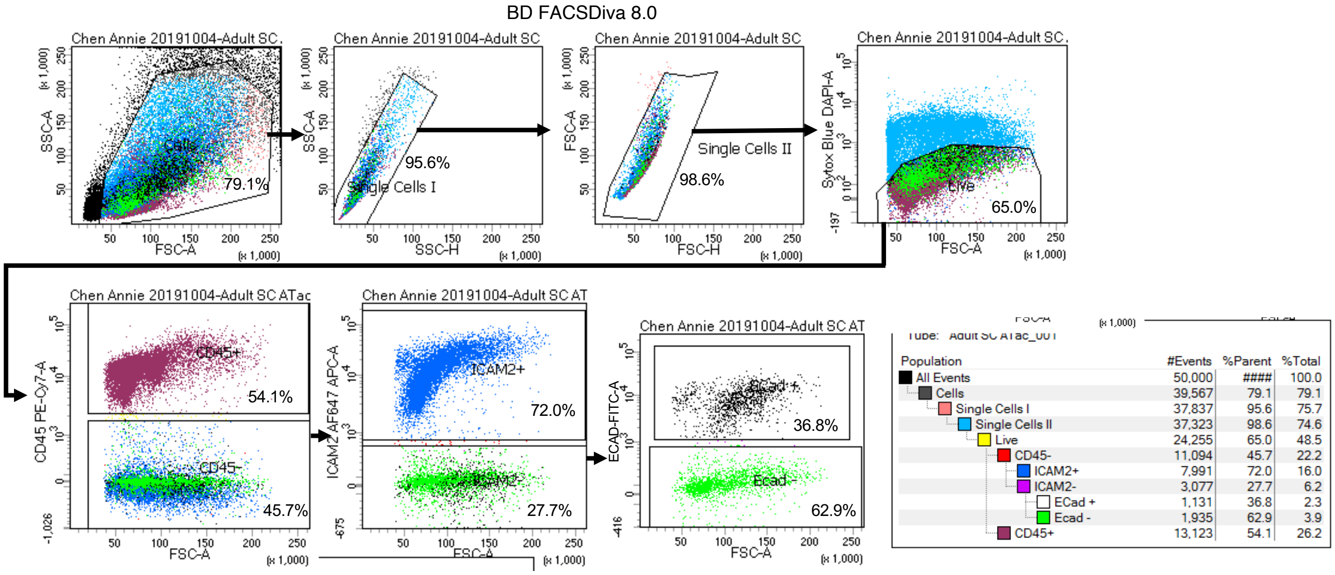

Population	#Events	%Parent	%Total
All Events	50,000	####	100.0
Cells	39,567	79.1	79.1
Single Cells I	37,837	95.6	75.7
Single Cells II	37,323	98.6	74.6
Live	24,255	65.0	48.5
CD45-	11,094	45.7	22.2
ICAM2+	7,991	72.0	16.0
ICAM2-	3,077	27.7	6.2
ECad +	1,131	36.8	2.3
Ecad -	1,935	62.9	3.9
CD45+	13,123	54.1	26.2

4. Sorting scheme for collecting epithelial cells for P15 littermate control and *Yap/Taz*$^{CKO/CKO}$; *Wnt3a*$^{Cre/+}$ mouse lungs for scRNA-seq. The example below is of sorting 10-wk mouse lungs for P15 littermate control lungs where the final ECAD+ gated epithelial cells for scRNA-seq.

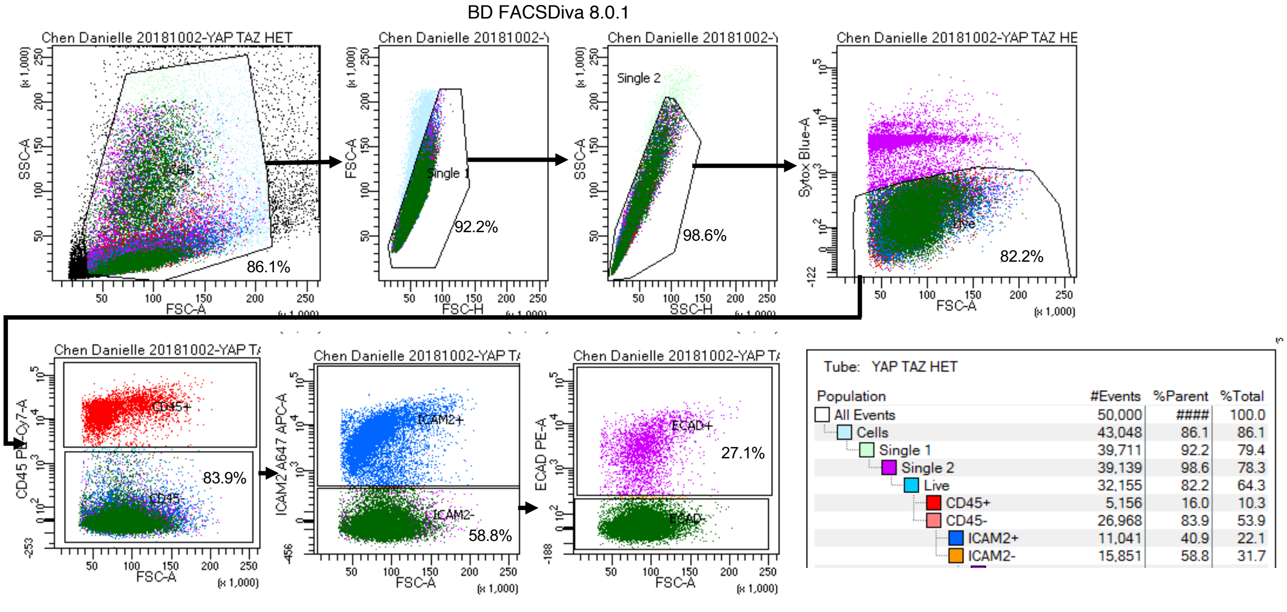

Population	#Events	%Parent	%Total
All Events	50,000	####	100.0
Cells	43,048	86.1	86.1
Single 1	39,711	92.2	79.4
Single 2	39,139	98.6	78.3
Live	32,155	82.2	64.3
CD45+	5,156	16.0	10.3
CD45-	26,968	83.9	53.9
ICAM2+	11,041	40.9	22.1
ICAM2-	15,851	58.8	31.7

5. Sorting scheme for collecting GFP positive cells for 10-wk littermate control and *Nkx2-1*$^{CKO/CKO}$; *Rosa*$^{Sun1GFP/+}$; *Rtkn2*$^{CreER/+}$ mouse lungs for ATAC-seq. The example below is from a control sample.

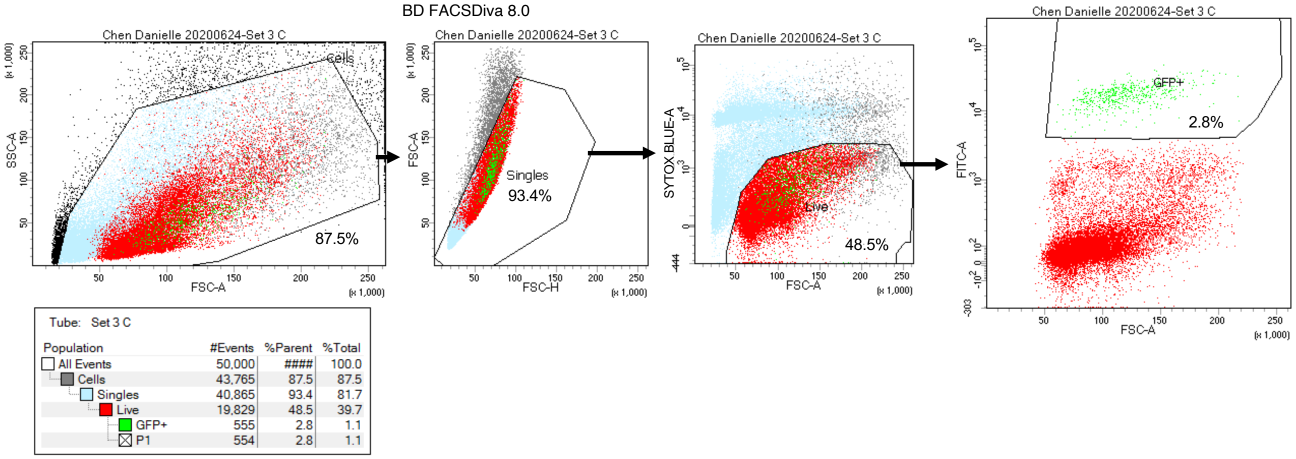

Population	#Events	%Parent	%Total
Tube: Set 3 C			
All Events	50,000	####	100.0
Cells	43,765	87.5	87.5
Singles	40,865	93.4	81.7
Live	19,829	48.5	39.7
GFP+	555	2.8	1.1
P1	554	2.8	1.1

6. Sorting scheme for collecting GFP positive cells for 10-wk littermate control and *Nkx2-1*$^{CKO/CKO}$; *Rosa*$^{Sun1GFP/+}$; *Sftpc*$^{CreER/+}$ mouse lungs for ATAC-seq. The example below is from a control sample.

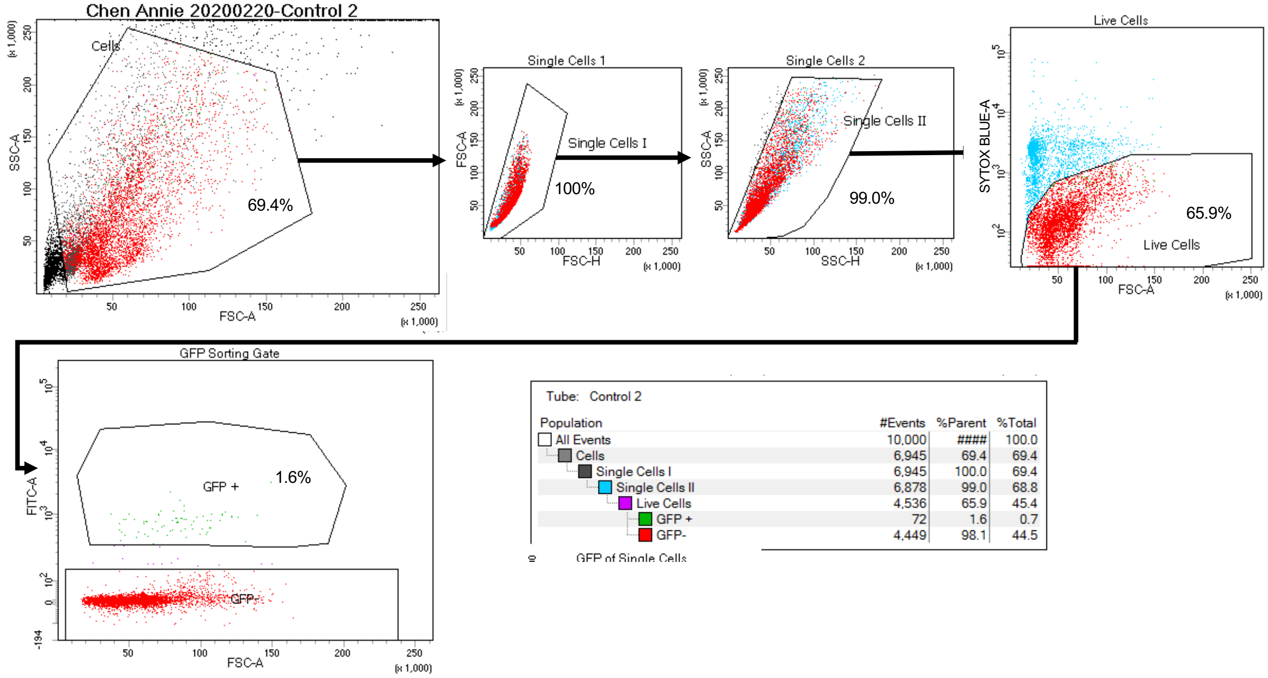

Population	#Events	%Parent	%Total
Tube: Control 2			
All Events	10,000	####	100.0
Cells	6,945	69.4	69.4
Single Cells I	6,945	100.0	69.4
Single Cells II	6,878	99.0	68.8
Live Cells	4,536	65.9	45.4
GFP +	72	1.6	0.7
GFP-	4,449	98.1	44.5
