# Supplementary material for: Differential chromatin binding of the lung lineage transcription factor NKX2-1 resolves opposing murine alveolar cell fates in vivo
Source: Nat Commun. 2021 May 4;12:2509. doi: 10.1038/s41467-021-22817-6 (PMC8096971; doi:10.1038/s41467-021-22817-6)

Source Data 3: Uncropped DNA gels for Supplementary Figure 3a. Relevant lanes are boxed. Positive control DNA (CreER) was used in place of a DNA ladder on the second gel.

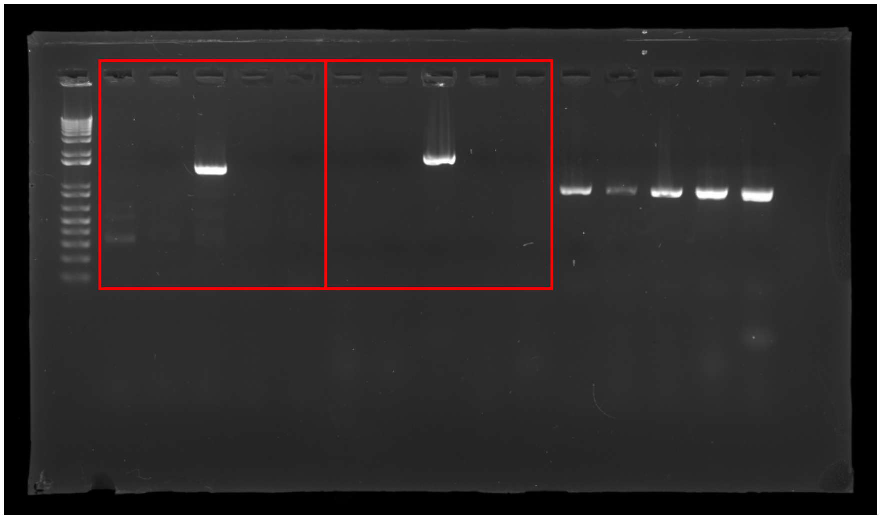

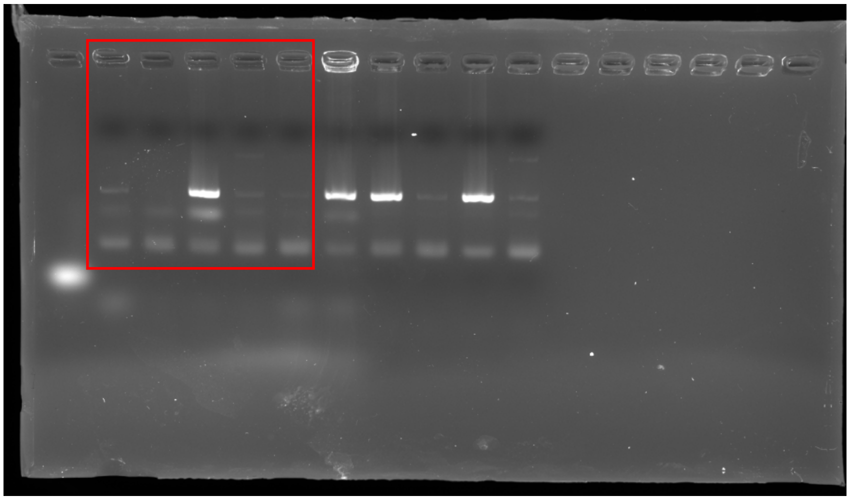

Supplement: Supplementary file 16 — Source Data [file 41467_2021_22817_MOESM16_ESM.zip › Source Data 3.pdf]
