# Supplementary material for: Differential chromatin binding of the lung lineage transcription factor NKX2-1 resolves opposing murine alveolar cell fates in vivo
Source: Nat Commun. 2021 May 4;12:2509. doi: 10.1038/s41467-021-22817-6 (PMC8096971; doi:10.1038/s41467-021-22817-6)

Source Data 4: Newborn Y/T^{Sox9} mutants (m) and littermate controls (c) from two litters received 3 mg tamoxifen at E15.5 and born one day overdue because tamoxifen interferes with pregnancy. All mutants are cyanotic and dead except for one that is gasping, a sign of respiratory stress.

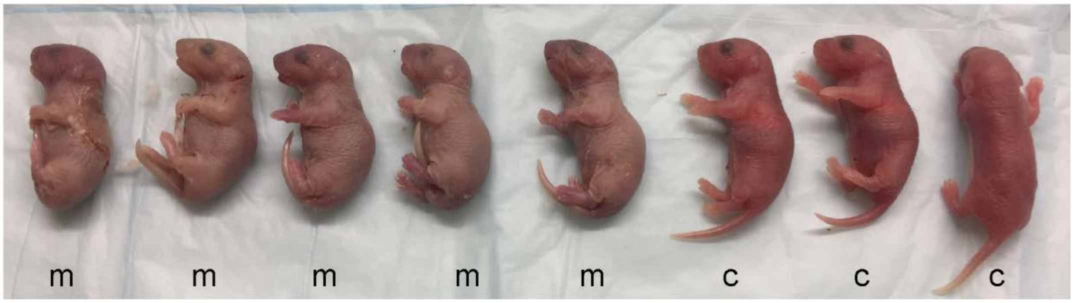

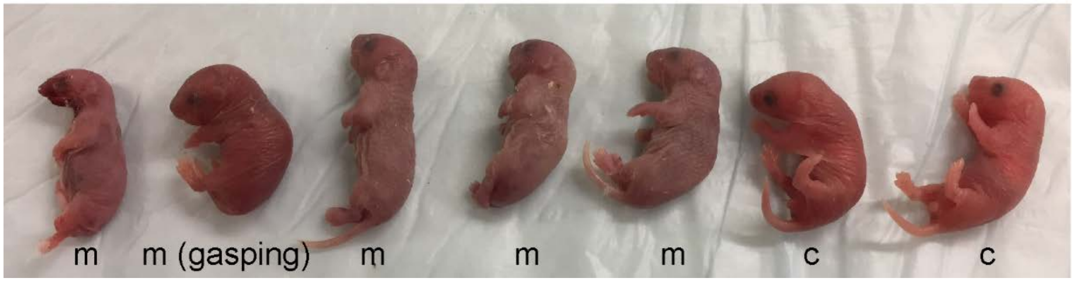

Supplement: Supplementary file 16 — Source Data [file 41467_2021_22817_MOESM16_ESM.zip › Source Data 4.pdf]
